# Linguistic Variation and Change in 250 Years of English Scientific Writing: A Data-Driven Approach

**DOI:** 10.3389/frai.2020.00073

**Published:** 2020-09-16

**Authors:** Yuri Bizzoni, Stefania Degaetano-Ortlieb, Peter Fankhauser, Elke Teich

**Affiliations:** ^1^Language Science and Technology, Saarland University, Saarbrücken, Germany; ^2^Digital Linguistics, Institut für Deutsche Sprache, Mannheim, Germany

**Keywords:** linguistic change, diachronic variation in language use, register variation, evolution of Scientific English, computational language models

## Abstract

We trace the evolution of Scientific English through the Late Modern period to modern time on the basis of a comprehensive corpus composed of the Transactions and Proceedings of the Royal Society of London, the first and longest-running English scientific journal established in 1665. Specifically, we explore the linguistic imprints of specialization and diversification in the science domain which accumulate in the formation of “scientific language” and field-specific sublanguages/registers (chemistry, biology etc.). We pursue an exploratory, data-driven approach using state-of-the-art computational language models and combine them with selected information-theoretic measures (entropy, relative entropy) for comparing models along relevant dimensions of variation (time, register). Focusing on selected linguistic variables (lexis, grammar), we show how we deploy computational language models for capturing linguistic variation and change and discuss benefits and limitations.

## 1. Introduction

The language of science is a socio-culturally firmly established domain of discourse that emerged in the Early Modern period (ca. 1500–1700) and fully developed in the Late Modern period (ca. 1700–1900). While considered fairly stable linguistically (cf.Görlach, 2001; Leech et al., 2009), the Late Modern period is a very prolific time when it comes to the formation of text types, with many of the registers we know today developing during that period—including the language of science (see Görlach, 2004 for a diachronic overview).

Socio-culturally, register diversification is connected to the growing complexity of modern societies, labor becoming increasingly divided with more different and increasingly specialized activities across all societal sectors^1^. Also, driven by science as well as early industry, standardization (e.g., agreements on weights and measures) and routinization of procedures become important issues. At the same time, enlightenment and the scientific and industrial revolutions support a general climate of openness and belief in technological advancement. In the domain of science, the eighteenth century is of course the epoch of encyclopedias^2^ but also that of the scientific academies which promoted the scientific method and distributed scientific knowledge through their publications. The two oldest scientific journals are the French *Journal des Sçavans* and the *Philosophical Transactions of the Royal Society of London*. At the beginning of publication (both started in 1665), the journals were no more than pamphlets and included articles written in the form of letters to the editor and reviews of scientific works (Gleick, 2010). Professionalization set in around the mid eighteenth century, as witnessed by the introduction of a reviewing process in the Royal Society (Moxham and Fyfe, 2018; Fyfe et al., 2019).

While there is a fair stock of knowledge on the development of scientific language from socio-cultural and historical-pragmatic perspectives (see section 2), it is less obvious what are the underlying, more general principles of linguistic adaptation to new needs of expression in an increasingly diversified and specialized setting such as science. This provides the motivation for the present research. Using a comprehensive diachronic corpus of English scientific writing composed of the Philosophical Transactions and Proceedings of the Royal Society of London [henceforth: Royal Society Corpus (RSC); Kermes et al., 2016; Fischer et al., 2020], we trace the evolution of Scientific English looking for systematic linguistic reflexes of specialization and diversification, yielding a distinctive “scientific style” and forming diverse sublanguages (sublanguage of chemistry, physics, biology etc.). In terms of theory, our work is specifically rooted in register linguistics (Halliday, 1985b; Biber, 1988) and more broadly in theories of language use, variation and change that acknowledge the interplay of social, cognitive and formal factors (e.g., Bybee, 2007; Kirby et al., 2015; Aitchison, 2017; Hundt et al., 2017). While we zoom in on the language of science, we are ultimately driven by the more general questions about language change: What changes and how? What drives change? How does change proceed? What are the effects of change? Thus, we aim at general insights about the dynamics of language use, variation and change.

In a similar vein, the methodology we present can be applied to other domains and related analysis tasks as well as other languages. Overall, we pursue an exploratory, data-driven approach using state-of-the-art computational language models (ngram models, topic models, word embeddings) combined with selected information-theoretic measures (entropy, relative entropy) to compare models/corpora along relevant dimensions of variation (here: time and register) and to interpret the results with regard to effects on language system and use. Since the computational models we use are word-based, words act as the anchor unit of analysis. However, style is primarily indicated by lexico-grammatical usage, so we investigate both the lexical and the grammatical side of words. While we consider lexis and grammar as intricately interwoven, in line with various theories of grammar (Halliday, 1985a; Hunston and Francis, 2000; Goldberg, 2006), for expository purposes, we here consider the lexico-semantic and the lexico-grammatical contributions to change separately.

The remainder of the paper is organized as follows. We start with an overview of previous work in corpus and computational linguistics in modeling diachronic change with special regard to register and style (section 2). In section 3 we introduce our data set (section 3.1) and elaborate on the methods employed (section 3.3). Section 4 shows analyses of diachronic trends at the levels of lexis and grammar (section 4.1), the development of topics over time (section 4.2) and paradigmatic effects of changing language use (section 4.3). Finally, we summarize our main results and briefly assess benefits and shortcomings of the different kinds of models and measures applied to the analysis of linguistic variation and change (section 5).

## 2. Related Work

The present work is placed in the area of language variation and change with special regard of social and register variation and computational models of variation and change (for overviews see Aragamon, 2019 for computational register studies and Nguyen et al., 2016 for computational socio-linguistics).

Regarding the language of science, there is an abundance of linguistic-descriptive work, including diachronic aspects, providing many valuable insights (e.g., Halliday, 1988; Halliday and Martin, 1993; Atkinson, 1999; Banks, 2008; Biber and Gray, 2011, 2016). However, most of the existing work is either based on text samples or starts from predefined linguistic features. Further, there are numerous studies on selected scientific domains, such as medicine or astronomy, e.g., Nevalainen (2006); Moskowich and Crespo (2012) and Taavitsainen and Hiltunen (2019), which work on the basis of fairly small corpora containing hand-selected and often manually annotated material. Typically, such studies are driven from a historical socio-linguistic or pragmatic perspective and focus on selected linguistic phenomena, e.g., forms of address (Taavitsainen and Jucker, 2003). For overviews on recent trends in historical pragmatics/socio-linguistics (see Jucker and Taavitsainen, 2013; Säily et al., 2017). Studies on specific domains, registers or text types provide valuable resources and insights into the socio-historical conditions of language use. Here, we build upon these insights, adding to it the perspective of general mechanisms of variation and change.

More recently, the diachronic perspective has attracted increasing attention in computational linguistics and related fields. Generally, diachronic analysis requires a methodology for comparison of linguistic productions along the time line. Such comparisons may range over whole epochs (e.g., systemic changes from early Modern English to Late Modern English), or involve short ranges (e.g., the issues of 1 year of The New York Times to detect topical trends). Applying computational language models to diachronic analysis requires a computationally valid method of comparison of language use along the time line, i.e., one that captures linguistic change if it occurs.

Different kinds of language models are suitable for this task and three major strands can be identified. First, a number of authors from fields as diverse as literary studies, history and linguistics have used simple ngram models to find trends in diachronic data using relative entropy (Kullback-Leibler Divergence, Jensen-Shannon Divergence) as a measure of comparison. For instance, Juola (2003) used Kullback-Leibler Divergence (short: KLD) to measure rate of linguistic change in 30 years of National Geographic Magazine. In more recent, large-scale analyses on the Google Ngram Corpus (Bochkarev et al., 2014; Kim et al., 2014) analyze change in frequency distributions of words within and across languages. Specifically humanistic research questions are addressed by e.g., Hughes et al. (2012) who use relative entropy to measure stylistic influence in the evolution of literature; or Klingenstein et al. (2014) who analyze different speaking styles in criminal trials comparing violent with non-violent offenses; or Degaetano-Ortlieb and Teich (2018) applying KLD as dynamic slider over the time line of a diachronic corpus of scientific text.

Second, probabilistic topic models (Steyvers and Griffiths, 2007) have become a popular means to summarize and analyze the content of text corpora, including topic shifts over time. In linguistics and the digital humanities, topic models have been applied to various analytic goals including diachronic linguistic analysis (Blei and Lafferty, 2006; Hall et al., 2008; Yang et al., 2011; McFarland et al., 2013). Here again, a valid method of comparing model outputs along the time line has to be provided. In our work, we follow the approach proposed in Fankhauser et al. (2016) using entropy over topics as a measure to assess topical diversification over time.

Third, word embeddings have become a popular method for modeling linguistic change, with a focus on lexical semantic change (e.g., Hamilton et al., 2016; Dubossarsky et al., 2017, 2019; Fankhauser and Kupietz, 2017). Word embeddings are weakly neural models that capture usage patterns of words and are used in a variety of NLP tasks. While well-suited to capture the summative effects of change (groups of words or whole vocabularies, see e.g., Grieve et al., 2016), the primary focus lies on lexis^3^. Other linguistic levels, e.g., grammar (Degaetano-Ortlieb and Teich, 2016, 2018; Bizzoni et al., 2019a), collocations (Xu and Kemp, 2015; Garcia and Garćia-Salido, 2019), or specific aspects of change, e.g., spread of change (Eisenstein et al., 2014), specialization (Bizzoni et al., 2019b) or life-cycles of language varieties (Danescu-Niculescu-Mizil et al., 2013), are only rarely considered. Once again, while word embeddings offer a specific model of language use, using them to capture diachronic change and to assess effects of change calls for adequate instruments for comparison along the time line. Here, we use the commonly applied measure of cosine distance for a general topological analysis of diachronic word embedding spaces; and we use entropy for closer inspection of specific word embeddings clusters to measure the more fine-grained paradigmatic effects of change.

In sum, in this paper we address some of the core challenges in modeling diachronic change by (a) looking at the *interplay* of different linguistic levels (here: lexis and grammar), (b) elaborating on the formation of style and register from a diachronic perspective, and (c) enhancing existing computational methods with explicit measures of linguistic change. Since we are driven by the goal of explanation rather than high-accuracy prediction (as in NLP tasks), qualitative interpretation by humans is an integral step. Here, micro-analytic and visual support are doubly important if one wants to explore linguistic conditions and effects of change. To support this, good instruments for human inspection and analysis of data are crucial—see, for instance, Jurish (2018) and Kaiser et al. (2019) providing visualization tools for various aspects of diachronic change, partly with interactive function (Fankhauser et al., 2014; Fankhauser and Kupietz, 2017); or Hilpert and Perek (2015)'s application of motion charts to the analysis of meaning change. We developed a number of such visualization tools made available as web applications for inspection of the Royal Society Corpus (cf. section 3).

## 3. Data and Methods

### 3.1. Data

The corpus used for the present analysis is the Royal Society Corpus 6.0 (Fischer et al., 2020). The full version is composed of the Philosophical Transactions and Proceedings of the Royal Society from 1665 to 1996. In total, it contains 295,895,749 tokens and 47,837 documents. Here, we use a version that is open-source under a creative commons license covering the period of 1665 to 1920. In terms of periods of English, this reflects the Late Modern period (1700–1900) plus a bit of material from the last decades of the Early Modern period (before 1700) as well as a number of documents from modern English. Altogether this open version contains 78,605,737 tokens and 17,520 documents.

Note that the RSC is not balanced, later periods containing substantially more material than earlier ones (see Table 1), which calls for caution regarding frequency effects. Other potentially interesting features of the corpus are that the number of different authors increases over time; so does the number of papers with more than one author.

**Table 1:** Size of RSC 6.0 by 50-year periods.

**Time**	**# Tokens**	**# Texts**
1665–1699	2,582,856	1,325
1700–1749	3,414,795	1,686
1750–1799	6,342,489	1,819
1800–1849	9,112,274	2,774
1850–1899	36,993,412	6,754
1900–1919	19,273,112	3,049

The documents in the corpus are marked up with meta-data including author, year of publication, text type and time period (1-, 10-, 50-year periods). The corpus is tokenized, lemmatized, annotated with part-of-speech tags and normalized (keeping both normalized and original word forms) using standard tools (Schmid, 1995; Baron and Rayson, 2008). The corpus is made available under a Creative Commons license, downloadable and accessible via a web concordance (CQPWeb; Hardie, 2012) as well as interactive visualization tools^4^.

### 3.2. Methods

There are two important a priori considerations regarding modeling linguistic change and variation. First, one of the key concepts in language variation is *use in context*. Apart from extra-linguistic, situational context (e.g., field, tenor, and mode; Quirk et al., 1985), intra-linguistic context directly impacts on linguistic choice, both syntagmatically (as e.g., in collocations) and paradigmatically (i.e., shared context of alternative expressions). Different computational models take into account different types of context and accordingly reveal different kinds of linguistic patterns. Topic models take into account the distribution of words in document context and are suitable to capture the field of discourse (see section 3.2.2 below). Plain ngram models take into account the immediately preceding words of a given word and can reveal syntagmatic usage patterns (see section 3.2.1 below). Word embeddings take into account left and right context (e.g., ± five words) and allow clustering words together depending on similar, surrounding contexts; thus, they are suited for capturing linguistic paradigms (see section 3.2.3 below).

Second, diachronic linguistic analysis essentially consists of *comparison of corpora* representing language use at different time periods. Computational language models being representations of corpora, the core task consists in comparing model outputs and elicit significant differences between them. Common measures of comparing language models are perplexity and relative entropy, typically used for assessing the quality or fit of a model by estimating the difference between models in bits using a log base. Here, we use the asymmetric version of relative entropy, Kullback-Leibler Divergence, to assess differences between language models according to time. An intimately related measure is entropy. Entropy considers the richness and (un)evenness of a sample and is a common means to measure diversity, e.g., the lexical diversity of a language sample (Thoiron, 1986). Here, we use entropy as a measure of diversification at two levels, the level of topics (field of discourse) and the level of paradigmatic word clusters, where greater entropy over time is interpreted as a signal of linguistic diversification and lower entropy as a signal of consolidated language use. The most basic way of exploring change in a given data set is to test whether the entropy over a simple bag-of-words model changes or not. For diversification to hold, we would expect the entropy to rise over time in the RSC, also because of the increase in size of the more recent corpus parts as well as in number of authors. As will be seen, this is not the case, entropy at this level being fairly stable (section 4.2).

#### 3.2.1. Ngram Based Models

To obtain a more fine-grained and linguistically informed overview of the overall diachronic tendencies in the RSC than possible with token ngrams, we consider lexical and grammatical usage separately using lemmas and part-of-speech (POS) sequences as modeling units. On this basis, models of different time periods (e.g., decades) are compared with the asymmetric variant of relative entropy, Kullback-Leibler Divergence (KLD; Kullback and Leibler, 1951); cf. Equation (1) where A and B here denote different time periods.

(1) $$\begin{array}{r} D\left(A||B\right)=\underset{i}{\sum }p\left(unit_{i}|A\right)log_{2}\frac{p\left(unit_{i}|A\right)}{p\left(unit_{i}|B\right)} \end{array}$$

KLD is a common measure for comparing probability distributions in terms of the number of additional bits needed for encoding when a non-optimal model is used. Applied to diachronic comparison, we obtain a reliable index of difference between two corpora A and B: the higher the amount of bits, the greater the diachronic difference. Also, we know which specific units/features contribute to the overall KLD score by their pointwise KLD. Thus, we can inspect particular points in time (e.g., by ranking features by pointwise KLD in 1 year) or time spans (e.g., by standard deviation across several years) to dynamically observe changes in a feature's contribution. This gives us two advantages over traditional corpus-based approaches: no predefined features are needed and results are more directly interpretable.

Apart from comparing predefined time periods with each other as is commonly done in diachronic corpus-linguistic studies (cf.Nevalainen and Traugott, 2012 for discussion), KLD can be used as a data-driven periodization technique (Degaetano-Ortlieb and Teich, 2018, 2019). KLD is dynamically pushed over the time line comparing past and future (or, as KLD is asymmetric, future vs. past). As we will show below, using KLD in this way allows detecting diachronic trends that are hard to see on a token level or with predefined, more coarse time periods. The granularity of diachronic comparison can be varied depending on the corpus and the analytic goal (year-, month-, day-based productions); again, no a priori assumptions have to be made regarding the concrete linguistic features involved in change other than selecting the linguistic level of comparison (e.g., lemmas, parts of speech). Hence, the method is generic and at the same time sensitive to the data.

#### 3.2.2. Topic Models

To obtain a picture of the diachronic development in terms of field of discourse—a crucial component in register formation—we need to consider the usage of words in the context of whole documents. To this end, we use topic models. We follow the overall approach of applying topic models to diachronic corpora mapping topics to documents (Blei and Lafferty, 2006; Steyvers and Griffiths, 2007; Hall et al., 2008; Yang et al., 2011; McFarland et al., 2013). The principle idea is to model the generation of documents with a randomized two-stage process: For every word *w*_*i*_ in a document *d* select a topic *z*_*k*_ from the document-topic distribution *P*(*z*_*k*_|*d*) and then select the word from the topic-word distribution *P*(*w*_*i*_|*z*_*k*_) . Consequently, the document-word distribution is factored as: $P\left(w_{i}|d\right)=\underset{k}{\sum }P\left(w_{i}|z_{k}\right)P\left(z_{k}|d\right)$. This factorization effectively reduces the dimensionality of the model for documents, improving their interpretability: Whereas *P*(*w*_*i*_|*d*) requires one dimension for each distinct word (tens of thousands) per document, *P*(*z*_*k*_|*d*) only requires one dimension for each topic (typically in the range of 20–100). To estimate the document-topic and topic-word distributions from the observable document-word distributions we use Gibbs-Sampling as implemented in mallet^5^.

To investigate topical trends over time, we average the document-topic distributions for each year *y*:

(2) $$\begin{array}{r} P\left(z_{k}|y\right)=1/n\underset{d_{j}\in y}{\sum }P\left(z_{k}|d_{j}\right) \end{array}$$

where *n* is the number of documents per year.

For further interpretation, we cluster topics hierarchically on the basis of the distance^6^ between their topic-document distributions (Equation 3).

(3) $$\begin{array}{r} P\left(d|z\right)=P\left(z|d\right)/\underset{j}{\sum }P\left(z|d_{j}\right) \end{array}$$

Topics that typically co-occur in documents have similar topic-document distributions, and thus will be placed close in the cluster tree.

To assess diachronic diversification in discourse field as a central part of register formation, we measure the entropy over topics (cf. Equation 4), and the mean entropy of topic-word distributions per time period.

(4) $$\begin{array}{r} H\left(P\left(.|y\right)\right)=-\underset{k}{\sum }P\left(z_{k}|y\right)log_{2}P\left(z_{k}|y\right) \end{array}$$

Note that all measures operate on relative frequencies per time period in order to control for the lack of balance in our data set (more recent periods contain considerably more data than earlier ones).

#### 3.2.3. Word Embeddings

Word embeddings (wes) capture lexical paradigms, i.e., sets of words sharing similar syntagmatic contexts. Word embeddings build on the principle underlying distributional semantics that it is possible to capture important aspects of the semantics of words by modeling their context (Harris, 1954; Lenci, 2008).

Here, we apply wes diachronically to explore the overall development of word paradigms in our corpus with special regard to register/sublanguage formation as well as scientific style. Using the approach and tools provided by Fankhauser and Kupietz (2017) we compute wes with a structured skip-gram approach (Ling et al., 2015). This is a variant of the popular Word2Vec approach (Mikolov et al., 2013). Word2Vec is a way of maximizing the likelihood of a word given its context, by training a *d* x *V* matrix where *V* is the vocabulary and *d* an arbitrary number of dimensions.

The goal of the algorithm is to maximize

(5) $$\begin{array}{r} L=\frac{1}{T}\underset{t\in T}{\sum }\underset{-c\leq j\leq c}{\sum }log\text{p}\left(w_{t+j}|w_{t}\right) \end{array}$$

where *T* is a text and *c* is the number of left and right context words to be taken into consideration. In short, the model tries to learn the probability of a word given its context, *p*(*w*_*o*_|*w*_*i*_). To this end, the model learns a set of weights that maximizes the probability of having a word in a given context. Such set of weights constitutes a word's embedding.

Usually, skip-gram considers a term's context as a bag-of-words. In Ling et al. (2015)'s variant, the order of the word context is also taken into consideration which is important to capture words with grammatical functions rather than lexical words only. For diachronic application, we calculate wes per time period (e.g., 1-/10-/50-year periods), where the first period is randomly initialized, and each subsequent period is initialized by the model for its preceding period. Thereby, wes are comparable across periods.

To perform analyses on our models, we then apply simple similarity measures commonly used in distributional semantics, where the similarity between two words is assessed by the cosine similarity of their vectors:

(6) $$\begin{array}{r} sim\left(w_{1},w_{2}\right)=cos\left(w_{1},w_{2}\right)=\frac{w_{1}w_{2}}{\left|w_{1}||w_{2}\right|} \end{array}$$

where *w*_1_ and *w*_2_ are the vectors of the two words taken into consideration, and |*w*| is a vector's norm. Alternatively, the semantic distance between words can be considered, which is the complement of their similarity:

(7) $$\begin{array}{r} dist\left(w_{1},w_{2}\right)=1-cos\left(w_{1},w_{2}\right) \end{array}$$

To detect the semantic tightness or level of clustering of a group of words (how semantically similar they are), one can thus compute the average cosine similarity between all the words in a group of words:

(8) $$\begin{array}{r} sim\left(V\right)=\frac{\sum_{w_{a}\in V}\sum_{w_{b}\in V}cos\left(w_{a},w_{b}\right)}{V^{2}} \end{array}$$

where *V* (vocabulary) is the group of words taken into consideration. Reversely, it is possible to compute the average distance of a group of words from another group of words by iterating the sums on two different sets.

To detect semantic shifts over time, one of the simplest and most popular approaches is that of computing the change of the cosine similarity between a group of pre-defined words in a chronologically ordered set of we spaces. As we will show, the we space of the RSC as a whole expands over time. At the same time, it becomes more *fragmented* and specific clusters of words become more densely populated while others disappear. We base such observations on an analysis of the word embeddings' topology using cosine similarity as explained above as well as entropy. For example, since the period under investigation witnesses the systematization of several scientific disciplines, we are likely to observe a narrowing of the meaning of many individual words—mainly technical terms—which would push them further away from one another. Similarly, for specific we clusters, we expect growth or decline, e.g., chemical terms explode in the late eighteenth century, pointing to the emergence of the field of chemistry with the associated technical language, or many Latin words disappear. Such developments can be measured by the entropy *H*(*P*(.|*w*)) over a given cluster around word *w*, by estimating the conditional probability of words *w*_*i*_ in the close neighborhood of word *w* as follows:

(9) $$\begin{array}{r} P\left(w_{i}|w\right)=sim\left(w,w_{i}\right)*freq\left(w_{i},w\right)/\left(\underset{k}{\sum }sim\left(w,w_{k}\right)*freq\left(w_{k},w\right)\right) \end{array}$$

where *w*_*k*_ ranges over all words (including *w*) with sufficient similarity (e.g., >0.6) to *w*. The neighbors are weighted by their similarity to the given word, thus, a word with many near neighbors and rather uniform distribution has a large entropy, indicating a highly diversified semantic field.

## 4. Analyses

Our analyses are driven by two basic assumptions: register diversification (linguistic variation focused on field of discourse) and formation of “scientific style” (convergence on specific linguistic usages within the scientific domain). We carry out three kinds of analysis on the Royal Society Corpus showing these two major diachronic trends at the levels of lexis and grammar (section 4.1), development of topic over time (section 4.2) as well as paradigmatic effects (section 4.3).

### 4.1. Diachronic Trends in Lexis and Grammar

We trace the overall diachronic development in the RSC considering both lexical and grammatical levels. Lexis is captured by lemmas and grammar by sequences of three parts of speech (POS). Using the data-driven periodization technique described in section 3.2.1 based on KLD, we dynamically compare probability distributions of lemma unigrams and POS trigrams along the time line.

**Figure 1:**
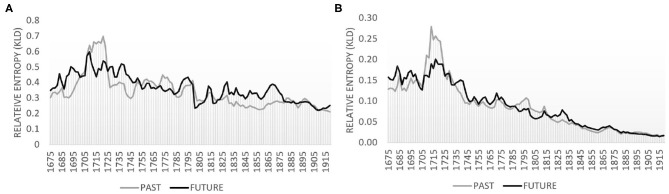
Relative entropy based on lemmas and part-of-speech trigrams with 2-year slider and 10-year past and future periods. **(A)** Lemmas. **(B)** Part-of-speech trigrams.

Figures 1A,B plot the temporal development for the lexical and the grammatical level, respectively. The black line visualizes relative entropy of the future modeled by the past, i.e., how well at a particular point in time the future can be modeled by a model of the past (here: 10 year slices). The gray line visualizes the reverse, i.e., how well the past is modeled by the future (again on 10-year slices). Peaks in the black line indicate changes in the future which are not captured by a model of the past, such as new terminology. Peaks in the gray line indicate differences from the opposite perspective, i.e., the future not encompassing the past, e.g., obsolete terminology. Troughs for both lines indicate convergence of future and past. A fairly persistent, low-level relative entropy indicates a period of stable language use.

Comparing the two graphs in Figure 1, we observe a particularly strong decreasing tendency for the grammatical level (see Figure 1B) and a slightly declining tendency at the lexical level with fairly pronounced oscillations of peaks and troughs (Figure 1A). Basically, peaks indicate innovative language use, troughs indicate converging use, the future being less and less “surprised” by the past. Thus, while grammatical usage consolidates over time, the lexical level is more volatile as it reacts directly to the pressure of expressing newly emerging things or concepts in peoples' (changing) domains of experience (here: new scientific discoveries). The downward trend at the grammatical level is a clear sign of convergence, possibly related to the formation of a scientific style; peaks at the lexical level signal innovative use and may indicate register diversification.

**Figure 2:**
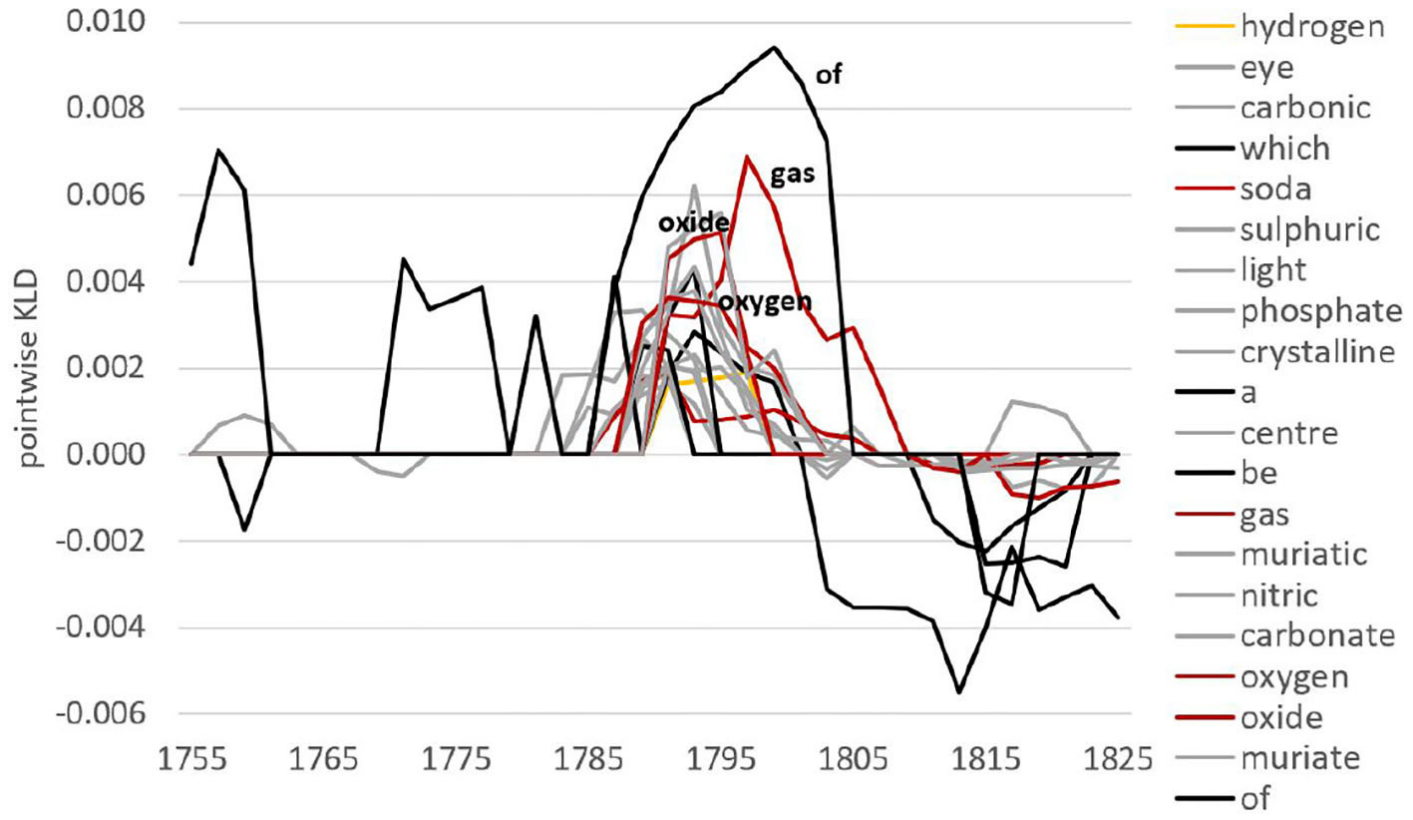
Pointwise relative entropy based on lemmas for the future model in 1791.

**Figure 3:**
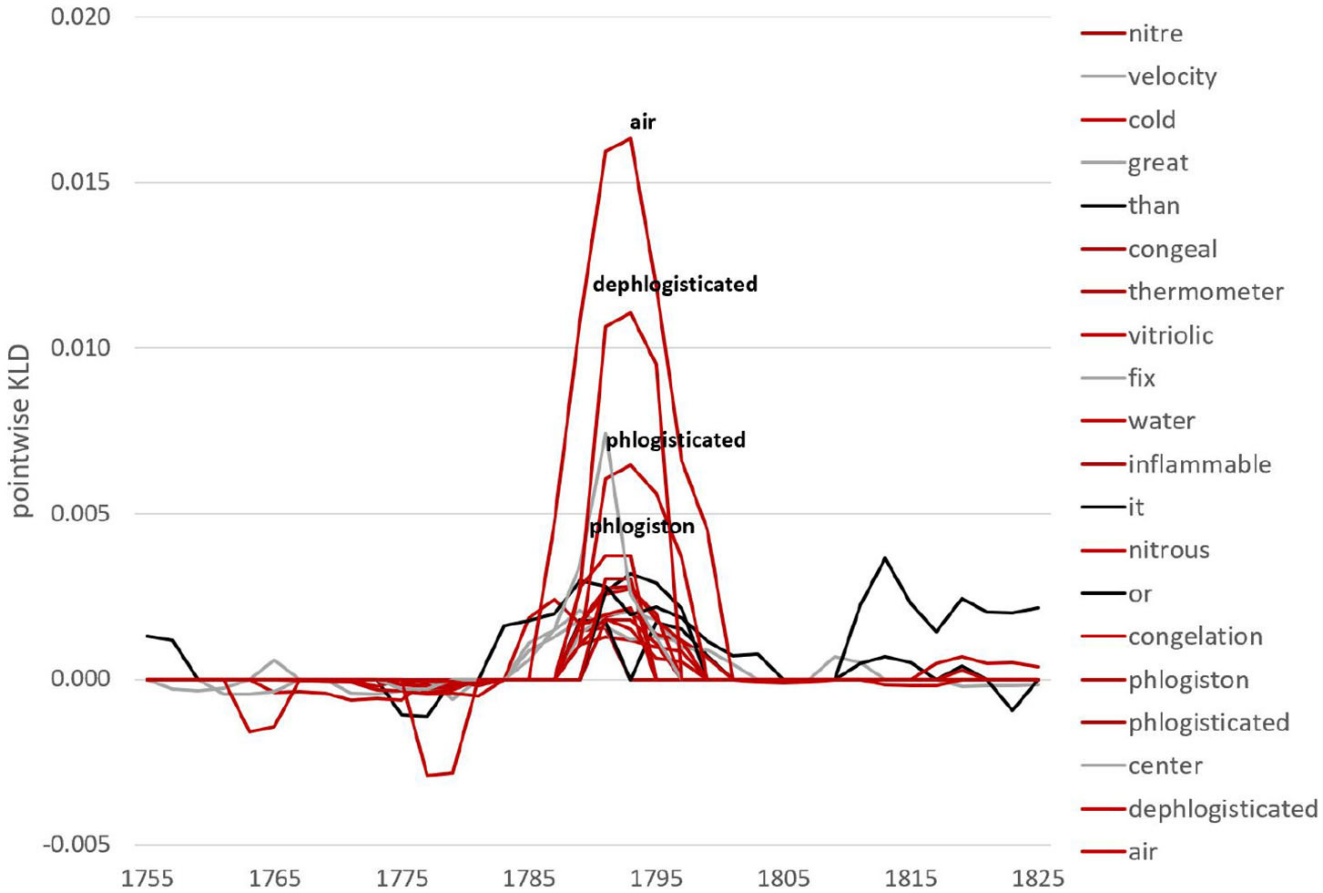
Pointwise relative entropy based on lemmas for the past model in 1791.

To investigate this in more detail, we look at specific lexical and grammatical developments. We use pointwise KLD (i.e., the contribution of individual features to overall KLD) to rank features. For example, there is a major increase in overall KLD around the 1790s at the lemma level. Considering features contributing to the highest peak in 1791 for the future model (black line), we see a whole range of words from the chemistry field around *oxygen* (see Figure 2). At the same time, we can inspect which features leave language use and contribute to an increase in KLD for the past model (i.e., features not well-captured by the future anymore). From Figure 3, we observe words related to *phlogiston* and experiments with air contributing to the formation of the oxygen theory of combustion (represented by Lavoisier, Priestley as well as Scheele). In fact, the oxygen theory replaced Becher and Stahl's 100-years old phlogiston theory, marking a chemical revolution in the eighteenth century—it is this shift of scientific paradigm that we encounter here in the RSC.

**Figure 4:**
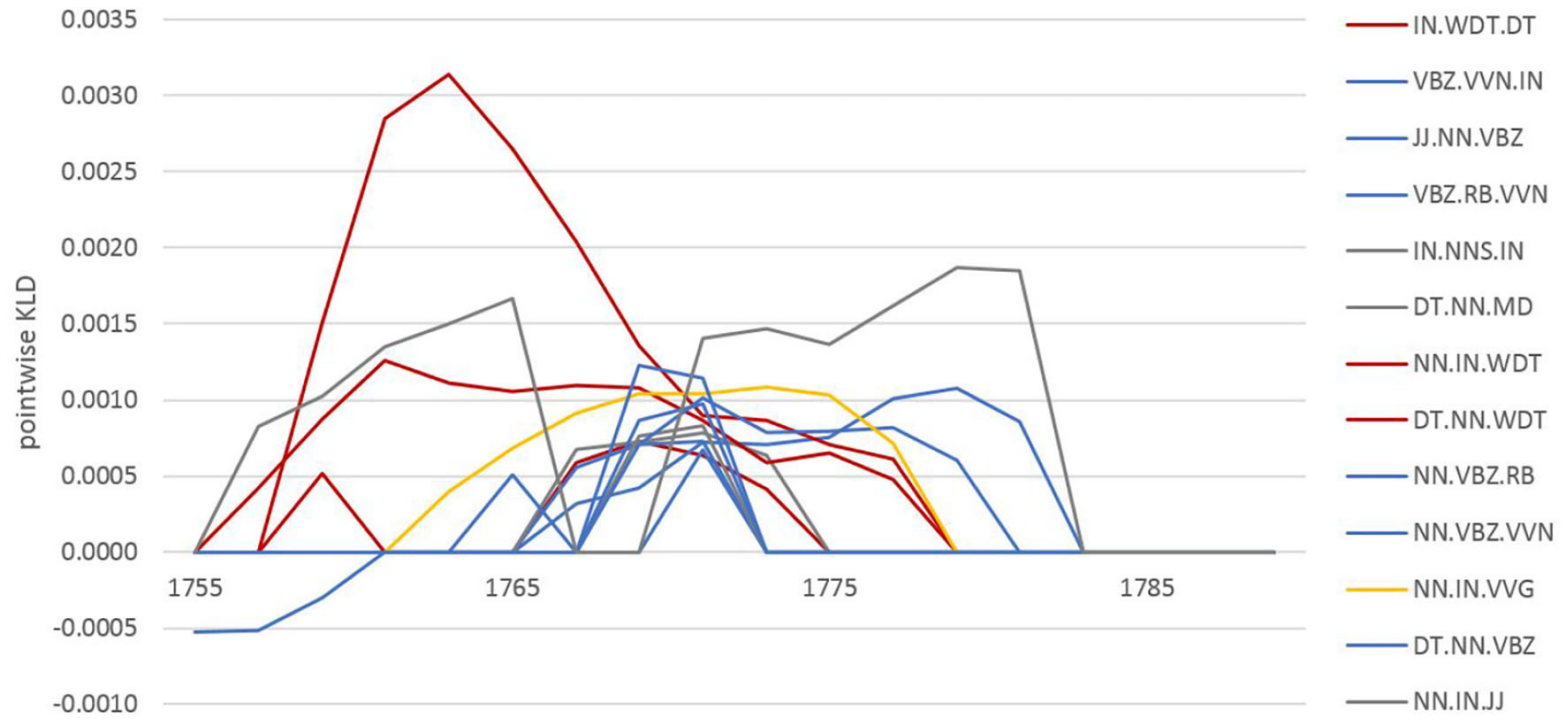
Pointwise relative entropy based on POS trigrams for the past model in 1771.

At the grammatical level, after a fairly high KLD peak in the early 1700's, there is a step-wise, steady decrease with only local, smaller peaks. As an example of a typical development at the grammatical level consider the features involved in the 1771 peak (see Figure 4). These are passive voice and relational verb patterns (e.g., noun-BE-participle as in *air is separated*; blue), nominal patterns with prepositions [e.g., indicating measurements such as the noun-preposition-adjective as in *the quantity of common (air)*; gray], gerunds (e.g., noun-preposition-*ing*verb, such as *method of making*; yellow), and relative clauses (e.g., determiner-noun-relativizer, such as *the air which/that*; red). While the contribution of these patterns to the overall KLD is high in 1771, it becomes zero for all of them by 1785—a clear indication of consolidation in grammatical usage pointing to the development of a uniform scientific style.

Regarding the lexical level, to verify that the observed tendencies point to significant diversification effects, we need to explore the systematic association of words with discourse fields. For this, we turn to topic models.

### 4.2. Diachronic Development of Discourse Fields

To analyse the development of discourse fields over time as the core component in register diversification, we trained a topic model with 30 topics^7^. Stop words were excluded and documents were split into parts of at most 5000 tokens each to control for largely varying document lengths.

Table 2 shows four of the 30 topics with their most typical words. Note that topics do not only capture the field of discourse (biology 3) but also genre (reporting), mode (formulae), or simply reoccurring boiler plate text (headmatter).

**Table 2:** Top five words for selected topics.

**REPORTING**	**HEADMATTER**	**BIOLOGY 3**	**FORMULAE**
great	vol	cells	equation
time	society	fig	equations
made	london	cell	function
found	author	tissue	form
account	part	nucleus	cos

**Figure 5:**
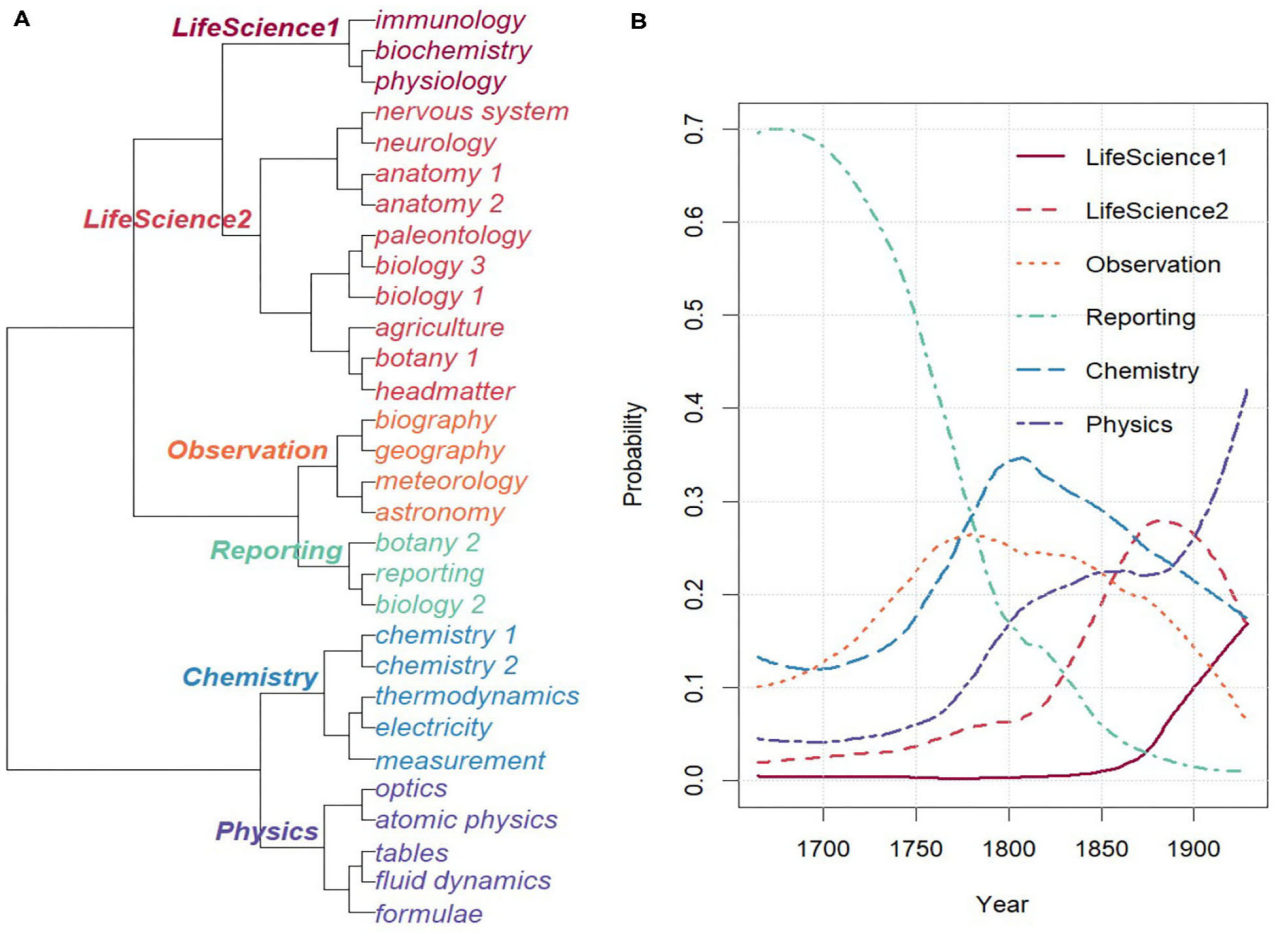
Overview on topics. **(A)** Topic hierarchy. **(B)** Combined topics over time.

Figure 5A displays the topic hierarchy resulting from clustering the topics based on the Pearson Distance between their topic-document distributions^8^. Labels for topics and topic clusters have been assigned manually, and redundant topics with very similar topic word distributions, such as biology, have been numbered through.

Figure 5B shows the probabilities of the combined topics over time. As can be seen, the first hundred years are dominated by the rather generic combined topic reporting, which covers around 70% of the topic space. Indeed, the underlying topic reporting makes for more than 50% of the topic space during the first 50 years. Starting in 1750, topics become more diversified into individual disciplines, indicating register diversification in terms of discourse field. In addition, in line with the analysis in section 3.1, we clearly see the rise of the chemistry topic around the 1790s.

**Figure 6:**
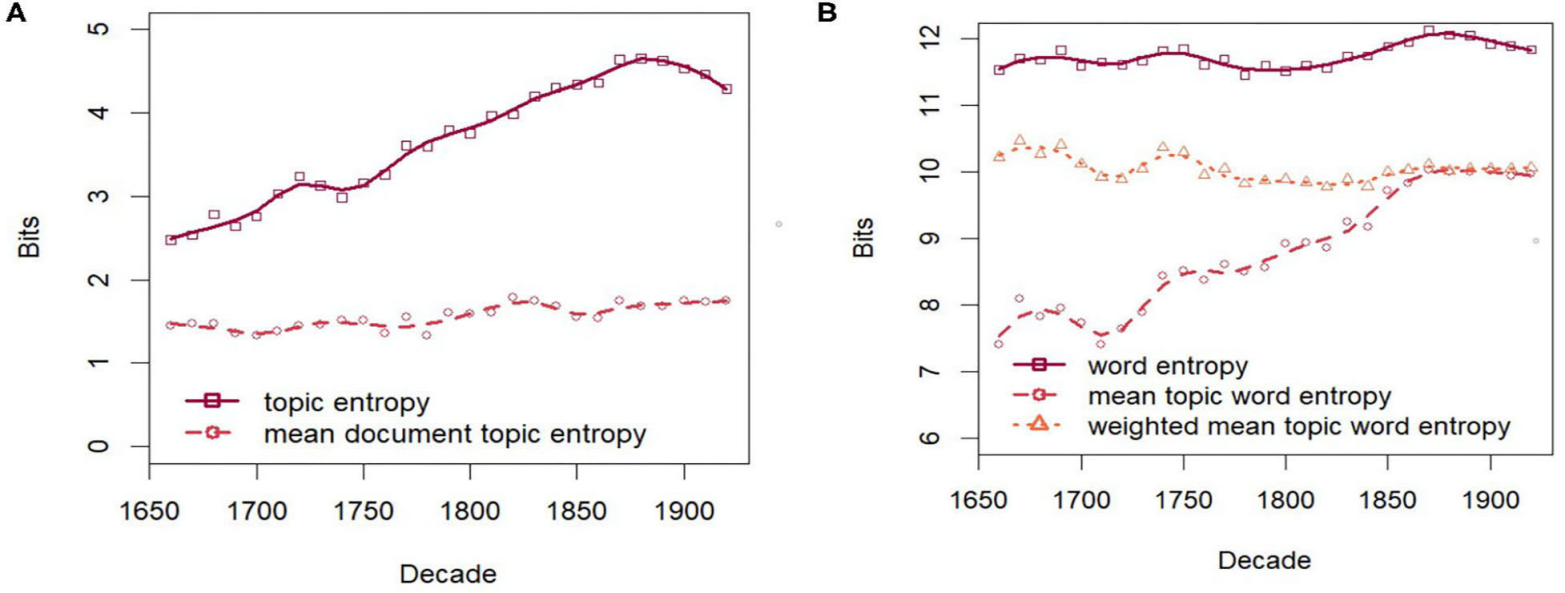
Entropies over time. **(A)** Entropy of topics. **(B)** Entropy of words.

As shown in Figure 6A diversification is evidenced by the clearly increasing entropy of the topic distribution over time. However, the mean entropy of the individual document-topic distributions remains remarkably stable, even though the mean number of authors per document and document length increase over time. Even the mean entropy weighted by document length (not shown) remains stable. This may be in part due to using asymmetric priors for the document-topic distributions, which generally skews them toward topics containing common words shared by many documents (Wallach et al., 2009), thus stabilizing the document-topic distributions over time.

**Figure 7:**
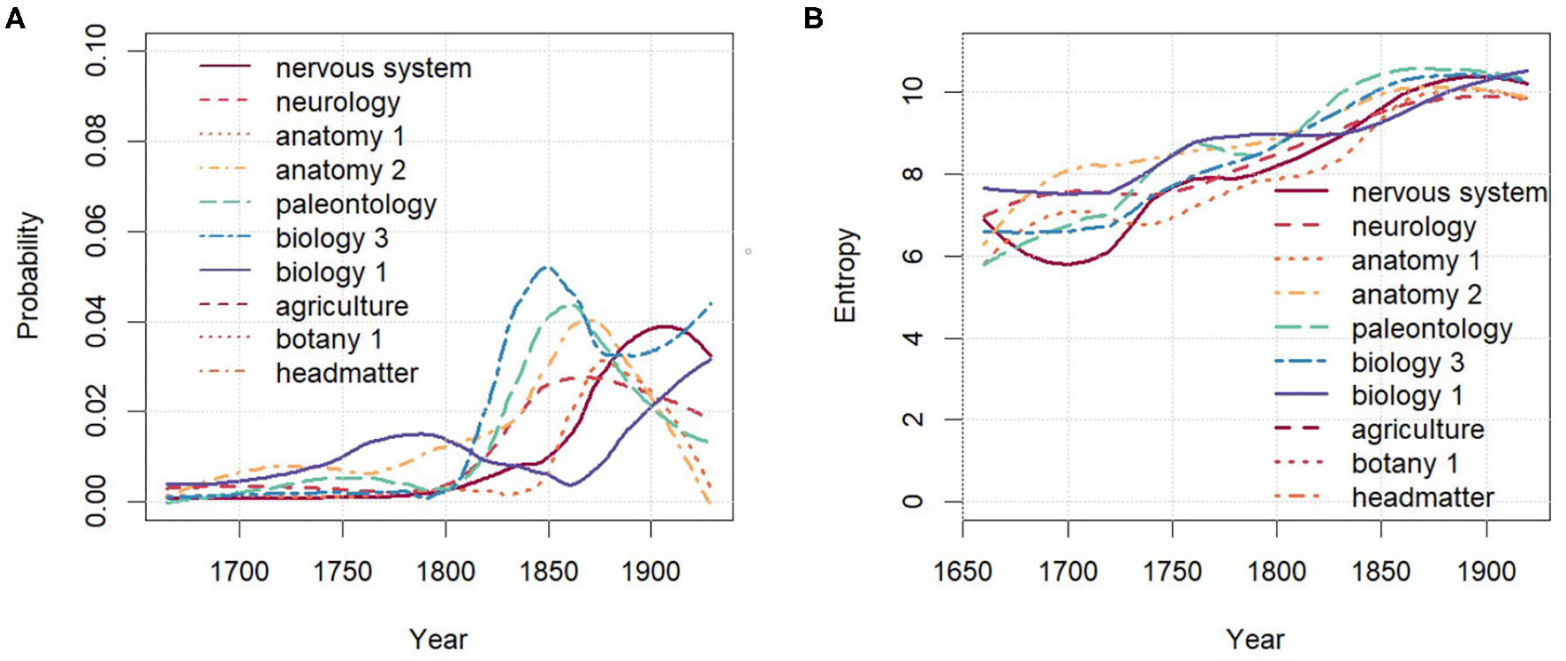
lifescience 2 over time. **(A)** Probability. **(B)** Entropy of topic word distributions.

Figure 6B shows the diachronic development of entropies at the level of words. The overall entropy of the unigram language model as well as the mean entropy of the topic word distributions weighted by the topic probabilities are also remarkably stable. However, the (unweighted) mean entropy of topic word distributions clearly increases over time. Indeed, due to the fairly high correlation of 0.81 (Spearman) between topic probability and the topic word entropy, evolving topics with increasing probability also increase in their word entropy, i.e., their vocabulary becomes more diverse. Figure 7 demonstrates this for the evolving topics in the group lifescience 2. All topics increase over time both in probability and entropy^9^. As will be seen in section 4.3, this trend is mirrored in the analysis of paradigmatic word clusters by word embeddings.

### 4.3. Paradigmatic Effects

To gain insights into the paradigmatic effects of the diachronic trends detected by the preceding analyses, we need to consider word usage according to syntagmatic context. To capture grammatical aspects as well (rather than just lexical-semantic patterns), we take word forms rather than lemmas as a unit for modeling and we do not exclude function words.

**Figure 8:**
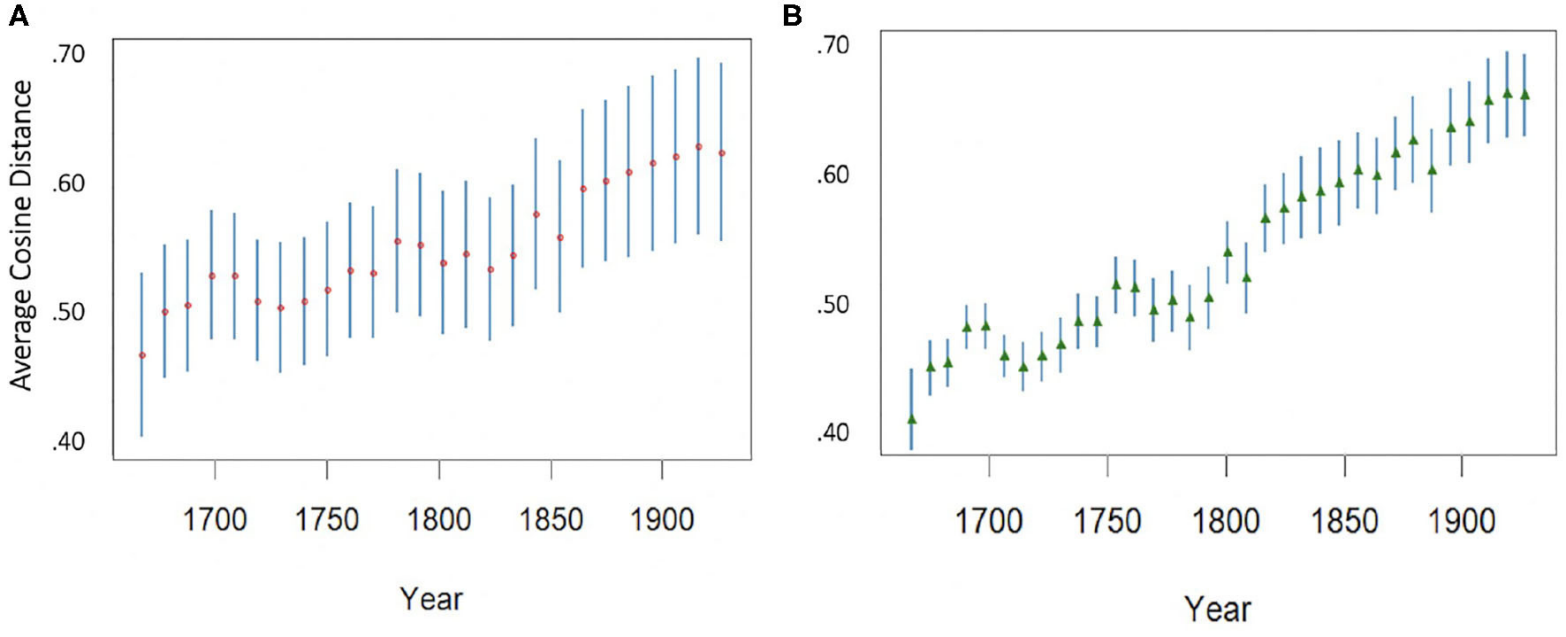
**(A)** Average distance and standard deviation of 2,000 randomly selected pairs of words. **(B)** Average distance from the whole vocabulary (mean and standard deviation) of 1,000 randomly selected words.

Based on the word embedding model as shown in section 3.2.3, we observe that the word embedding space of the RSC grows over time both in terms of *vocabulary size* and in terms of *average distance* between words. While a growing vocabulary can be interpreted in many ways, it is more informative to look at the increase in average distance between words. Here, not every term grows apart from all other terms (in fact, many pairs of words get closer through time) but when we take two random terms the average distance between them is likely to increase—see Figure 8: (A) shows the diachronic trend for the distance between 2,000 randomly selected pairs of words and (B) for the distance of 1,000 randomly selected words from the rest of the vocabulary. The words were selected among those terms that appear at least once in every decade. In both cases, the trend toward a growing distance is clearly visible.

Given that wes are based on similarity in context, this means that overall, words are used increasingly in different contexts, a clear sign of diversification in language use. For example, the usage of *magnify* and *glorify* diverges through the last centuries resulting in a meaning shift for *magnify* which becomes more associated with the aggrandizing effects of optical lenses while *glorify* remains closer to its original sense of elevating or making glorious. If we look for these two words in the we space, what we see is, in fact, a progressive decrease of the distributional similarity between them: for example, in 1860 their cosine distance is 0.48, while in 1950 it has gone up to 0.62. The nature of their nearest neighbors also diverges: *magnify* increasingly shows specialized, optic-related neighborhoods (*blood-globule* in 1730, *object-lens* in 1780, *eyeglass* in 1810) while the neighbors of *glorify* remain more mixed (mainly specific but non-technical verbs, such as *bill, reread, ingratiate*, with low similarity). Finally, their movement with respect to originally close neighbors is also consistent: e.g., the distance between *glorify* and *exalt* does not change between 1860 and 1920, while *magnify* appears to move away and back toward *exalt* through the decades and is more than 25 degrees further from it in 1920 than in 1670 (from 0.45 to 0.70).

To provide another example, a similar evolution is apparent for *filling* and *saturating*: their distance grows from 0.37 in 1700 to 0.65 in 1920, a difference of almost 30 degrees. In the same lapse of time, the distance between *saturating* and *packing* goes from 0.27 to 0.70. Actually, the meaning of *saturating* was originally closer to that of *satisfying* and *packing*: its usage as a synonym of *imbuing*, and its technical sense in chemistry are more recent, and have progressively drawn the word's usage apart from that of *filling*.

As noted above, we observe an overall expansion of the we space. To test whether this expansion is not a simple effect of the increase of frequency and number of words in each decade, we select a set of function words which exhibit stable frequency and should not change in usage over time (e.g., the functions of *the, and*, and *for* did not change in the period considered). If the expansion we observe is due to raw frequency effects, function words should drift apart from each other at a similar rate as content words. This appears not to be the case. As shown in Table 3, if we compare the group of function words to a group of randomly selected content words, such as verbs and nouns, we can see that the distances between the elements of such group grow much faster than the distances between function words. Purely functional words drift apart considerably less than words having a lexical meaning, indicating that the latter are probably causing most of the lexical expansion. Thus, words having a proper lexical meaning grow apart much faster *on average* than words having a purely functional role.

**Table 3:** Average cosine distance between function words vs. 2,000 randomly selected content words in the first and last decade of RSC 6.0 Open.

**Group**	**Full vocabulary**		**Persistent vocabulary**	
	**1670**	**1920**	**1670**	**1920**
Function words	0.44	0.51	0.46	0.47
Content words	0.45	0.70	0.44	0.63

*To account for the constantly updated vocabulary of scientific terminology, we present both the results for all words in each decade (Full Vocabulary) and for only those words that appear in every decade (Persistent Vocabulary)*.

**Figure 9:**
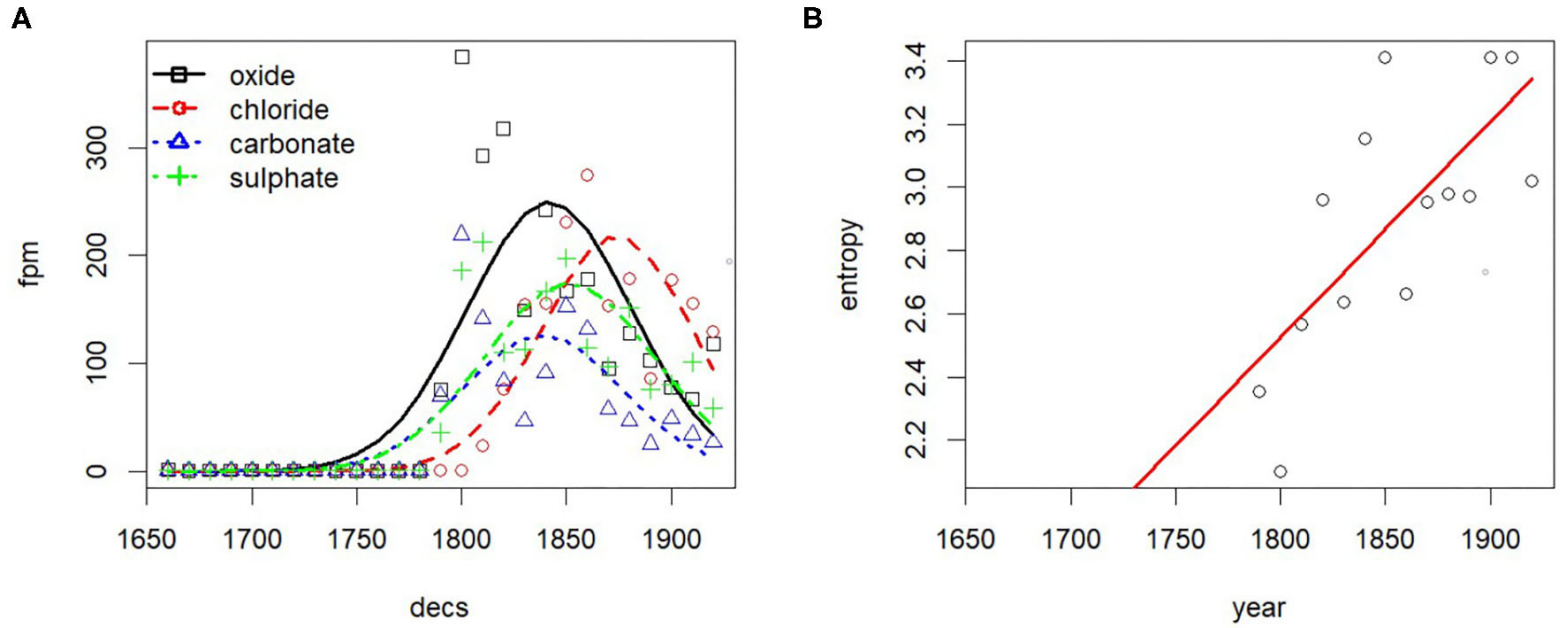
Entropy increase on specific we clusters signals terminological diversification. **(A)** Relative frequency. **(B)** Entropy.

This behavior is not consistent with a raw frequency effect, or with the side effects of changes in the magnitude of training data. It looks like the distributional profile of words is, on average, growing more distinct in this specific corpus. And this does not happen only for new vocabulary, created *ad hoc* for specific contexts: even when we factor out the changes in lexicon and we consider only those words that appear in every decade (Persistent Vocabulary in Table 3), the effect is still visible. This interpretation is supported when we inspect the entropy on specific we clusters over time. We consider two cases: increasing and decreasing entropy on a cluster, the former signaling lexical diversification, the latter signaling converging linguistic usage. For instance, coming back to the field of chemistry, we observe increasing entropy in particular clusters of content words: see Figure 9 for an example, showing (A) relative frequency of selected terms denoting chemical compounds and (B) entropy on the we cluster containing those terms (radius of cosine similarity > 0.6).

**Figure 10:**
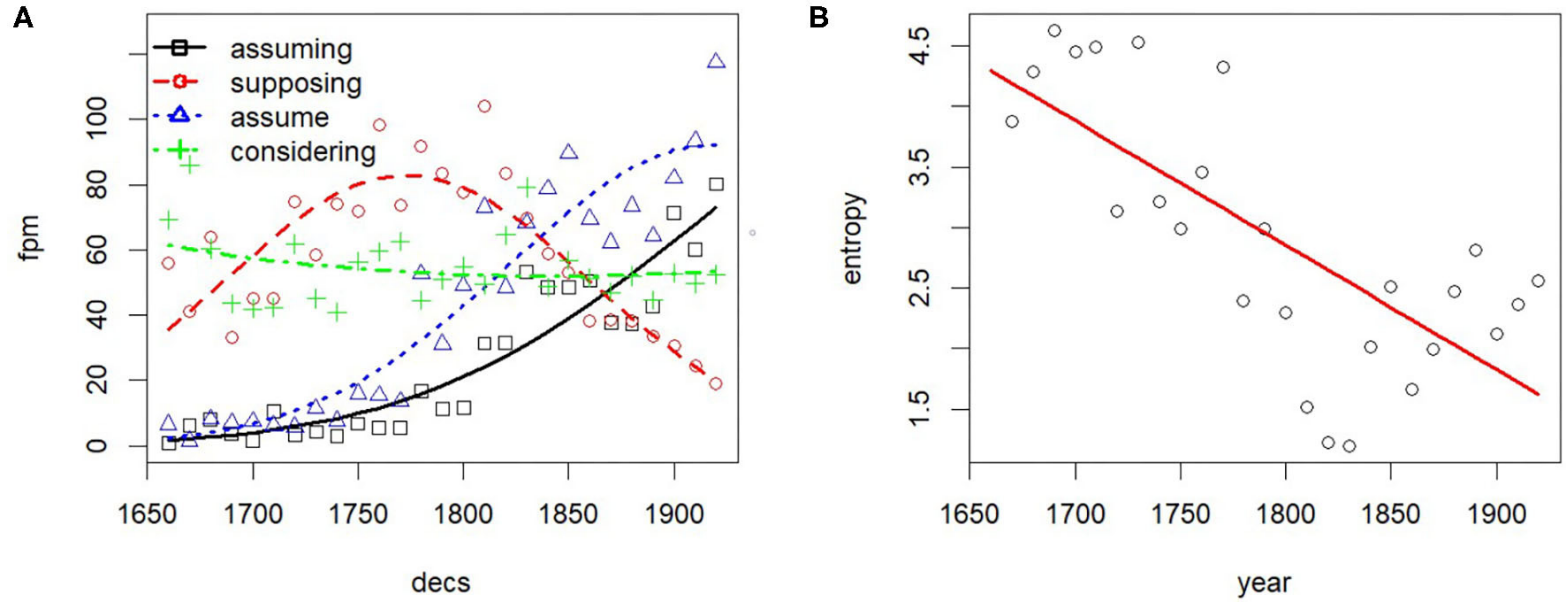
Entropy decrease on specific we clusters signals convergence in usage. **(A)** Relative frequency. **(B)** Entropy.

As an example of the opposite trend, i.e., decreasing entropy, consider the use of *ing*-forms which diversify according to the analysis above shown for *filling* and *saturating*, i.e., they spread to different syntagmatic contexts. In the example in Figure 10, the terms in the cluster containing *assuming* exhibit a skewed frequency over time with decreasing entropy, reflecting in this case stylistic convergence, i.e., the tacit agreement on using particular linguistic forms rather than others. In particular, *assuming* has 30 close neighbors (including *supposing, assume, considering*) in the first decade, but only 13 close neighbors in the last decade, with *assuming, assume* dominating by frequency.

The effect of stylistic convergence on the reduction of the cluster entropy of *assuming* is visible also through a cursory look at some corpus concordances. Uses of *assuming* in the sense of “adopting” disappear (see example 1). Over time, *assuming* comes to be used increasingly at the beginning of sentences (example 2), the dominant use being the non-finite alternative to a conditional clause (*If we assume a/the/that*.). In terms of frequency, the dominant choice in the cluster is *assume*, presumably as a short form of *let us/let's assume* (example 3), a usage that is often associated with mathematical reasoning.

1. *No notice is taken of any effervescence or discharge of air while it was assuming this color* (Cavendish, 1786).
2. *Assuming a distribution of light of the form when x is the distance along the spectrum from the center of the line, the half breadth is defined as the distance in which the intensity is reduced to half the maximum* (Strutt, 1919).
3. *Assume any three points a, b, c in the surface, no two of which are on one generator, [.]* (Gardiner, 1867).

## 5. Summary and Future Work

We have explored patterns of variation and change in language use in Scientific English from a diachronic perspective, focusing on the Late Modern period. Our starting assumption was that we will find both traces of diversification in terms of discourse field, thus pointing to register formation, as well as convergence in linguistic usage as indicator of an emerging scientific style. As a data set we used 250+ years of publications of the Royal Society of London [Royal Society Corpus (RSC), Version 6.0 Open].

We have elaborated a data-driven approach using three kinds of computational language models that reveal different aspects of diachronic change. Ngram models (both lemma and POS-based) point to an overall trend of consolidation in linguistic usage. But the lexical level dynamically oscillates between high peaks marking lexical innovation and lows marking stable linguistic use, where the peaks typically reflect new scientific discoveries or insights. At the grammatical level, we observe similar tendencies but at a much lower level and rate and the consolidation trend is much more obvious. Inspecting the specific grammatical patterns involved, we find that they mark what we commonly refer to as “scientific style,” such as relational and passive clauses or specific nominal patterns for hosting terminology.

To investigate further the tendencies at the level of words, we have looked at aggregations of words from two perspectives—how words group together to form topics (development of fields of discourse as the core factor in register formation) and how specific words group together to form paradigms based on their use in similar contexts. Diversification is fully born out from both perspectives with glimpses of consolidation as well. Analysis on the basis of a diachronic topic model shows that topics diversify over time, indexed by increasing entropy over topic/word distributions, a clear signal of register formation. Analysis on the basis of diachronic word embeddings reveals that the overall paradigmatic organization of the vocabulary changes quite dramatically: the lexical space expands overall and it becomes more fragmented, the latter being a clear signal of diversification in word usage. Here, bursts of innovation are shown by increasing entropy on specific word clusters, such as terms for chemical compounds, mirroring the insights from lemma-based analysis with KLD. Also, patterns of convergence (confined uses of words) as well as obsolescence (word uses leaving the language) are shown by decreasing entropy on particular word clusters, such as the cluster of *ing*-forms. Taken together, we encounter converging evidence of diversification at different levels of analysis; and at the same time we find signs of linguistic convergence as an overarching trend—an emerging tacit agreement on “how to say things”, a “scientific style.”

In terms of methods, we have elaborated a data-driven methodology for diachronic corpus comparison using state-of-the-art computational language models. To analyze and interpret model outputs, we have applied selected information-theoretic measures to diachronic comparison. Relative entropy used as a data-driven periodization technique provides insights into overall diachronic trends. Entropy provides a general measure of diversity and is applied here to capture diversification as well as converging language use for lexis (word embeddings) overall and discourse fields (topic models) in particular.

In future work, we will exploit more fully the results from topic modeling and the word embeddings model of the RSC. For instance, we want to systematically inspect high and low-entropy word embedding clusters to find more features marking expansion (vs. obsolescence) and diversification (vs. convergence). Also, annotating the corpus with topic labels from our diachronic topic model will allow us to investigate discipline-specific language use (e.g., chemistry) and contrast it with “general” scientific language (represented by the whole RSC) as well as study the life cycles of registers/sublanguages. Especially interesting from a sociocultural point of view would be to trace the spread of linguistic change across disciplines and authors (e.g., Did people adopt specific linguistic usages from famous scientists?). Finally, we would like to contextualize our findings from an evolutionary perspective and possibly devise predictive models of change. Our results seem to be in accordance not only with our intuitive understanding of the evolution of science but also with evolutionary studies on vocabulary formation (e.g., Smith, 2004) showing how populations using specialized vocabularies are more likely to develop and take over when the selective ratio is pure efficacy in information exchange.

## Data Availability Statement

The Royal Society Corpus (RSC) 6.0 Open is available at: https://hdl.handle.net/21.11119/0000-0004-8E37-F (persistent handle). Word embedding models of the RSC with different parameter settings including visualization are available at: http://corpora.ids-mannheim.de/openlab/diaviz1/description.html.

## Author Contributions

YB curated the analyses on word embeddings showing the diachronic expansion of the scientific semantic space and lexical-semantic specialisation. SD-O carried out the analysis on diachronic trends in lexis and grammar using data-driven periodization and elaborating on features' contribution to change. PF trained the word embeddings and the topic models and designed and implemented the entropy-based diachronic analysis. ET provided the historical background and was involved in hypothesis building and interpretation of results.

## Conflict of Interest

The authors declare that the research was conducted in the absence of any commercial or financial relationships that could be construed as a potential conflict of interest.

## Footnotes

^1^An example in point are production and experimentation, which used to be carried out hand in hand in the workshops of alchemists and apothecaries but were separated later on, also physically, with experimentation becoming a scientific activity carried out in dedicated laboratories (Burke, 2004; Schmidgen, 2011).

^2^For example, the publication of the famous *Encyclopédie ou Dictionnaire raisonné des sciences, des arts et des métiers* (1751–1765).

^3^For more comprehensive overviews on computational approaches to lexical semantic change see Tahmasebi et al. (2018) and on diachronic word embeddings see Kutuzov et al. (2018).

^4^RSC 6.0 Open: https://hdl.handle.net/21.11119/0000-0004-8E37-F.

^5^http://mallet.cs.umass.edu

^6^We use Pearson distance, which consistently results in more intuitive hierarchies than Jensen-Shannon Divergence.

^7^For the corpus at hand, a smaller number of topics leads to conflated topics, a larger number to redundant topics.

^8^Clustering by Jensen-Shannon Divergence results in a less intuitive hierarchy.

^9^A similar correlation between probability and entropy can be observed in other rising topic groups.
